# Soybean and fish oil mixture increases IL-10, protects against DNA damage and decreases colonic inflammation in rats with dextran sulfate sodium (DSS) colitis

**DOI:** 10.1186/1476-511X-9-68

**Published:** 2010-07-08

**Authors:** Karina V Barros, Roberta AN Xavier, Gilclay G Abreu, Carlos AR Martinez, Marcelo L Ribeiro, Alessandra Gambero, Patrícia O Carvalho, Claudia MO Nascimento, Vera LF Silveira

**Affiliations:** 1 Departamento de Fisiologia, Universidade Federal de São Paulo, São Paulo, SP, Brazil; 2 Multidisciplinary Research Unit, São Francisco University Medical School, Bragança Paulista, SP, Brazil; 3 Clinical Pharmacology and Gastroenterology Unit, São Francisco University Medical School, Bragança Paulista, SP, Brazil; 4 Departamento de Ciências Biológicas, Universidade Federal de São Paulo, Campus Diadema, SP, Brazil

## Abstract

It was investigated whether dietary polyunsaturated fatty acids (PUFA) could influence colonic injury, tissue DNA damage, cytokines and myeloperoxidase activity (MPO) and plasma corticosterone in DSS-induced colitis rats. Male weaning Wistar rats were fed for 47 days with an AIN-93 diet with control (C), fish (F) or a mixture of fish and soybean oil (SF). The colitis was induced from day 36 until day 42 by 3% DSS in drinking water. On day 48, blood samples were collected for corticosterone determination. The distal colon was excised for histological analysis and to quantify the cytokine (IL-4, IL-10 and INF-γ), MPO and DNA damage. The disease activity index (DAI) was recorded daily during colitis induction. The DAI, MPO, histological analyses showed decreases only in the SF group compared with the C group. IL-10 was increased and DNA damage was reduced in the groups F and SF, and an inverse correlation between these variables was found. There were no differences in corticosterone, IFN-γ and IL-4 levels. Soybean and fish oil mixture may be effective in improving colonic injury and DNA damage, and it could be an important complementary therapy in UC to reduce the use of anti-inflammatory drugs and prevent colorectal cancer.

## Introduction

Ulcerative colitis (UC) is an inflammatory bowel disease (IBD) characterized by recurrent episodes of colonic inflammation and tissue regeneration[1]. Although the pathogenesis of UC has not been entirely elucidated, the chronic relapsing inflammation has a multifactorial etiology. UC can be caused by an exaggerated immune response to the intestinal flora in the context of genetic predisposition [2,3] that can be attributed, at least in part, to an imbalance between effector T cells (Teff) and regulatory T cells (Treg)[4].

In IBD, there is increased synthesis and release of pro-inflammatory mediators, such as eicosanoids, platelet activating factor, reactive oxygen species (ROS), nitrogen metabolites, chemokines and mainly cytokines [5] that have been associated with disease severity, activity and remission [2].

Active episodes of UC are characterized by mucosal injury, increased vascular permeability, infiltration of neutrophilic polymorphonuclear, leukocytes, disruption of extracellular matrix and epithelial cell damage where the synthesis and release of ROS, triggered mainly by neutrophils, can mediate cell and tissues injury [6].

Patients with IBD are at increased risk of developing colorectal cancer, and the inflammation has been associated with neoplastic changes through production of pro-inflammatory cytokines and ROS[7]. Both mediators activate nuclear transcription factor-kB (NF-kB), inducible nitric oxide synthesis, and cyclooxygenase-2-related signaling pathways, which may retard or suppress apoptosis in intestinal epithelial cells and modulate angiogenesis [8]. ROS, the cellular consequences of oxidative stress, may cause DNA oxidation, resulting in damage to all four bases and the deoxy-ribose-molecule [9,10]. Chronic inflammation in the colonic mucosa caused by increased and continuous exposure of ROS promotes oxidative DNA damage of the epithelial cells, triggering the appearance of genetic mutations and initiating colorectal carcinogenesis [10,11].

Disturbances of fatty acid status may relate to metabolic consequences of IBD [12], and nutrition and dietary factors can modulate immune function. Dietary fatty acids such as omega 3 (w-3) polyunsaturated fatty acids (PUFA) can exert an anti-inflammatory effect reducing pro-inflammatory cytokines production [4].

The main purpose of this study was to examine the effect of diets enriched with fish oil, soybean oil and fish plus soybean oil mixture on markers of colonic injury, cytokines (IL-4, IL-10 and IFN), MPO activity, corticosterone levels and DNA damage in colon of rats with experimental UC induced by dextran sulfate sodium (DSS). Colitis induced by DSS is widely used due to the advantages of simplicity, degree of lesion uniformity, and leukocytes infiltration [13]. DSS is also an experimental model for oxidative stress [11,14].

## Materials and methods

### Animals and diet treatments

Eighteen male Wistar rats (28-30 days) were obtained from the Center for the Development of Experimental Models in Medicine and Biology at the Federal University of São Paulo. They were kept under controlled light conditions (12:12 h light-dark cycle with lights on at 07:00 A.M.) and temperature conditions (24 ± 1°C) with free access to food and water. The animals were separated into three groups (n = 6 per group) and received, for 47 days, one of three diets: control (C group), fish (F group) or soybean-fish (SF group) diet. All of the experiments reported were previously reviewed and approved by Institutional Ethics Committee for Experimental Research (n° 01588/07).

The diets were prepared according to the recommendations of the American Institute of Nutrition. The standard AIN-93 [15] G (8% fat until 2 month) and M (5% fat after 2 month) diets contained the same amount of protein, carbohydrates and lipids. The only difference between the diets was the source of lipids: 100% of soybean oil (source of *w*-6 PUFA) in the C group, 100% of fish oil (source of *w*-3 PUFA) in the F group and a mixture of 50% of soybean oil and 50% of fish oil in the SF group. We obtained soybean oil and fish oil from Brazilian producers. The detailed compositions of the diets are presented in Table 1, and the fatty acid profile of each diet is presented in Table 2.

**Table 1:** Composition of the experimental diets according AIN-93^15^

		Diet (g/100 g)	
	
Ingredient	Control	Fish	Soybean/Fish
Casein^+^	20.0 (14.0)	20.0 (14.0)	20.00 (14.0)
Corn starch*	62.0 (71.1)	62.0 (71.1)	62.00 (71.1)
Cellulose*	5.0 (5.0)	5.0 (5.0)	5.0 (5.0)
Mineral mix AIN-93^@^	3.5 (3.5)	3.5 (3.5)	3.5 (3.5)
Vitamin mix AIN 93^@^	1.0 (1.0)	1.0 (1.0)	1.0 (1.0)
Choline bitartrate*	0.25 (0.25)	0.25 (0.25)	0.25 (0.25)
L-cystina*	0.3 (0.18)	0.3 (0.18)	0.3 (0.18)
Butylhydroquinone*	0014 (0.007)	0.014 (0.007)	0.014 (0.007)
Fish oil^#^	0	8.0 (5.0)	4.0 (2.5)
Soybean oil^&^	8.0 (5.0)	0	4.0 (2.5)

The first number refers to the growth diet (AIN-93 G), and the number in parentheses refers to the maintenance diet (AIN-93 M). ^+^casein was obtained from Sinth. *L-cystine, corn starch, butylhydroquinone, cellulose and choline bitartrate were obtained from Viafarma, Brazil. Soybean oil^&^from Lisa, Brazil and fish oil from Campestre, Brazil. Mineral mix^@ ^and vitamin mix^@ ^AIN-93 were supplied by Rhoster Ind. and Com. LTDA.

**Table 2:** Fatty acids composition of the experimental diets

Percentage of total fatty acids			
**Fatty acids (%)**	**Control**	**Fish**	**Soybean/Fish**

14:0	0.6	8.9	2.3
16:0	10.2	20.8	12.9
18:0	3.2	5.5	3.9
16:1 (*n*-7)	1.6	8.4	4.3
18:1 (*n*-9)	20.5	9.8	15.2
18:2 (*n*-6)	49.4	4.8	35.6
18:3 (*n*-3)	4.9	0.8	2.4
20:4 (*n*-6)	Nd	Nd	Nd
20:5 (*n*-3)	Nd	16.2	8.2
22:6 (*n*-3)	Nd	14.9	6.4
Ni	9.6	9.9	8.8
Total SAFA	14.0	35.2	19.1
Total MUFA	22.1	18.2	19.5
Total PUFA	54.3	36.7	52.6
w-6 PUFA	49.4	4.8	35.6
w-3 PUFA	4.9	31.9	17.0
w-6:w-3 PUFA	10:1	1:6	2:1

SAFA, saturated fatty acids; MUFA, monounsaturated fatty acids; PUFA, polyunsaturated fatty acids; Nd, not detected, Ni, Not identified.

### Induction of colitis, samples collection and procedures

Colitis was induced in all animals from day 36 to day 42 with 3% DSS (wt/v, prepared daily, mol wt 5.000-Fluka BioChemika) put in the drinking water.

Animal body weight, presence of gross blood in the feces and stool consistency were recorded daily for each rat from day 35 to day 47. These parameters were each assigned a score according to the criteria proposed by Cooper *et al *[16], which was used to calculate a daily mean disease activity index (DAI). Food and water consumption was also recorded daily during this period.

On day 47, the rats, which were food deprived for 24 h, were anesthetized (1:1 xilazine-ketamine). Blood samples were collected by decapitation for plasma corticosterone determination (trisodium citrate, as anticoagulant). The distal colon was immediately excised, rinsed with phosphate buffered saline (PBS), weighed, and its length was measured under a constant load (2 g). The distal colon was longitudinally opened and subsequently divided into four segments: 2 cm to histological analysis (immediately fixed in 10% formaldehyde), 0.5 cm to DNA damage detection (maintained in a fixative solution described below), 0.5 cm to myeloperoxidase (MPO) activity determination, 0.5 cm to fatty acids composition and 6.0 cm to cytokines (IL-4, IL-10 and INF-g) measurements. All samples were stored at -80°C, except the histological analysis samples.

### Histological analysis

A cross-section of the distal colon (2 cm) was fixed in 10% paraformaldehyde solution. Afterwards, it was cut into small fragments, dehydrated through an ethanol series (70%-100%), cleared in xylol and embedded in paraffin. The fragments were sliced into 5 μm thick sections and stained with hematoxylin-eosin. Histological evaluation was done by a pathologist who was blinded to the experimental groups, and it was based on the intensity of mononuclear and polymorphonuclear infiltrates in the lamina propria, crypt dilation, cellular destruction and mucosal ulceration. Histopathological changes were graded according to the degree of inflammation using the following scale: absent (0), light (1), moderate (2) and intense (3), and the numbers represented the inflammation score (IS). Results were expressed as mean values of IS ± standard error of the mean (SEM) for each experimental group.

### Fatty acids composition of diets

For total lipid extraction, diets samples were homogenized in chloroform and methanol (2:1 v/v) followed by the addition of an aqueous solution of KCL [17]. The chloroform layer was dried under N_2_, and the total extract was converted into methyl esters of fatty acids using BF3 methanol, according to the method suggested by the American Oil Chemist's Society [18]. The methyl esters were diluted in hexane and analyzed by gas chromatography using a CHROMPACK^® ^chromatographer (model CP 9001) with a flame ionization detector and a CP-Sil 88 capillary column (Chrompak, WCOT Fused Silica 59 mm × 0.25 mm). The detector temperature was 280°C, and the injector temperature was 250°C. The initial temperature was 180°C for 2 minutes (min), programmed to increase 10°C per min up to 210°C and held for 30 min. The carrier gas used was hydrogen at a flow rate of 2.0 mL.min^-1^. The identification of the fatty acids was done comparing the retention times of the sample components with authentic standards of fatty acid esters injected under the same conditions. Fatty acid composition, as a percent of total acid weight, was calculated using area counts of the chromatogram.

### Measurement of colon cytokine and plasma concentration

Tissue samples were homogenized in 3.5 mL PBS solution and centrifuged at 1200 rpm for 10 minutes. Supernatants were transferred into clean Eppendorf tubes and stored at -80 °C. The concentration of INF-γ, IL-4 and IL-10 were measured by enzyme-linked immunosorbent assay technique using commercially available kits purchased from R&D Systems.

The plasma corticosterone concentration was quantified by the fluorimetric method [19].

### Myeloperoxidase Activity in the Colon

Tissues colon samples (0.5 cm) obtained from the distal colon were homogenized in 0.5% (w/v) hexadecyltrimethylammonium bromide in 50 mM potassium phosphate buffer, pH 6.0. For the myeloperoxidase (MPO) assay, 50 μL of each sample were added to 200 μL of o-dianisidine solution (0.167 mg/mL o-dianisidine dihydrochloride, 0.0005% hydrogen peroxide in 50 mM phosphate buffer, pH 6.0) immediately prior to reading the change in absorbance at 460 nm over 5 minutes using a microplate reader (Multiscan MS, Labsystems, Helsinki, Finland).

### Comet Assay

The Comet assay detects DNA damage (strand breaks and alkali-labile sites) at the individual cell level. Cells from distal colon samples were isolated as described below. The biopsies were pooled and incubated with 5.5 mg proteinase K (Sigma-Aldrich, St. Louis, MO, USA) and 3 mg collagenase (Invitrogen Life Technologies, Grand Island, NY, USA) in 3 mL of Hank's balanced salt solution (HBSS; Invitrogen) for 45 min at 37°C to liberate the cells; the cells were then re-suspended in 10 mL of HBSS. The resulting suspensions were centrifuged at 750 g for 5 min, and the supernatant was discarded. Since high leukocyte content could lead to a bias in the levels of DNA damage from colon cells, leukocyte contamination was assessed in the cell suspensions. Aliquots of 100 mL were dropped onto a slide, fixed with acetone, and stained with hematoxylin and eosin. The slides were analyzed by blinded examiners for leukocyte levels.

### Cell Viability

The Comet assay should be performed only on samples having a cell viability of more than 75%. Therefore, cell viability was determined using the fluorescein-diacetate (FDA)/ethidium bromide (EtBr; Sigma-Aldrich, St. Louis, MO, USA) assay. Briefly, a fresh staining solution was prepared containing 30 mL FDA in acetone (5 mg/mL), 200 mL EtBr in PBS (200 mg/mL), and 4.8 mL PBS (Invitrogen). The single cell suspension (25 mL) was then mixed with 25 mL of the staining solution, spread onto a slide and covered with a coverslip. Viable cells appeared fluorescent-green, whereas red-stained nuclei indicated dead cells. At least 200 cells were counted per sample.

### Determination of DNA Damage

The alkaline Comet assay was performed on the single cell suspensions, according to Singh *et al*.[20], but with some modifications. Briefly, 15 mL of the single cell suspension (~ 2 × 10^4 ^cells) were mixed with molten 0.5% low-melting-point agarose (Promega Co. Madison, WI, USA) and spread on agarose-precoated microscope slides. The slides were immersed overnight at 4°C in freshly prepared cold lysing solution (2.5 M NaCl, 100 mM EDTA, 10 mM Tris, 2% sodium salt N-Lauryl sarcosine, pH 10, with 1% Triton X-100 and 10% DMSO; all from Sigma-Aldrich). Subsequently, the cells were exposed to alkaline buffer (1 mM EDTA and 300 mM NaOH, pH ~13.4) at 4°C for 40 min to allow DNA unwinding and expression of alkali-labile sites. Electrophoresis was then conducted in the same solution at 4°C for 20 min using 25 V and 300 mA. After incubation, the slides were washed in cold PBS; lysis, denaturation and electrophoresis were then performed in the same manner as described above. After electrophoresis, the slides were neutralized (0.4 M Tris, pH 7.5), stained with 40 mL EtBr (20 mg/mL) and analyzed with a fluorescence microscope (Eclipse E400; Nikon, Melville, NY, USA), using an image analysis system (Komet 5.5; Kinetic Imaging, Nottingham, UK). Two hundred randomly selected cells (100 from each of two replicate slides) were evaluated from each sample, and the mean of the Olive tail moment DNA was determined. Tail moment (TM) is defined as the product of DNA in the tail, and the mean distance of migration in the tail is calculated by multiplying tail intensity/sum comet intensity by the center of gravity of the tail - peak position. A higher percentage of tail DNA signifies a higher level of DNA damage.

#### Statistical analysis

Data were expressed as means ± standard error of the mean (SEM). Statistical analysis was performed by one-way analysis of variance (ANOVA) followed by the Bonferroni's post hoc test for multiple comparisons. Significance level was set at p < 0.05. To verify the correlation between two variables, Pearson's correlation coefficients (r) were used.

## Results

### Fatty acids composition of diets and food intake

Table 2 shows the fatty acid composition of diets. The levels of monounsaturated fatty acids (MUFA) were not different among the diets. The saturated fatty acid (SAFA) was higher in the F group as compared with the C and SF groups. In the SF diet, in which fish oil was mixed with soybean oil, there was no excess of SAFA like there was in the Fish diet. In relation to PUFA, the F group had a lower amount compared with the C and SF groups; however, the main difference among the diets was the w-6:w-3 PUFA ratio. The C group showed a w6:w3 ratio that was similar to western diets (10:1) while the F group had a ratio (1:6) with an excess of w-3 PUFA. In the SF group, a balanced ratio (2:1) was observed.

No difference in food intake was observed among the three groups (data no shown).

### Body weight before and after colitis induction, disease activity index (DAI), colon length, inflammation score (IS) and corticosterone

Animals were monitored from weaning, and their weight was evaluated once a week before induction and daily after colitis induction. Up to day 36, there was no difference in weight among the groups; however, after colitis induction, groups SF and F presented with body weight increases compared with the C group (Table 3).

**Table 3:** Values indicate body weight (g) before and during induction of colitis by DSS

	Before induction of colitis					During induction of colitis	
**Days**	**7**	**14**	**21**	**28**	**35**	**42**	**47**

**Control**	129.83 ± 3.13	167.49 ± 2.19	209.41 ± 3.50	244.96 ± 3.26	286.25 ± 2.58	265.82 ± 5.88	278.39 ± 7.81
**Fish**	140.70 ± 5.05	172.33 ± 5.29	224.53 ± 8.07	264.47 ± 10.74	301.98 ± 4.14	290.36 ± 9.04*	311.69 ± 5.84*
**Soybean/Fish**	129.57 ± 4.08	168.79 ± 3.84	210.01 ± 4.00	250.23 ± 4.57	289.88 ± 5.28	301.58 ± 2.06*	314.86 ± 6.60*

Values represent means ± SEM, n = 6 rats; * Different (p < 0.05) from Control group.

**Figure 1:**
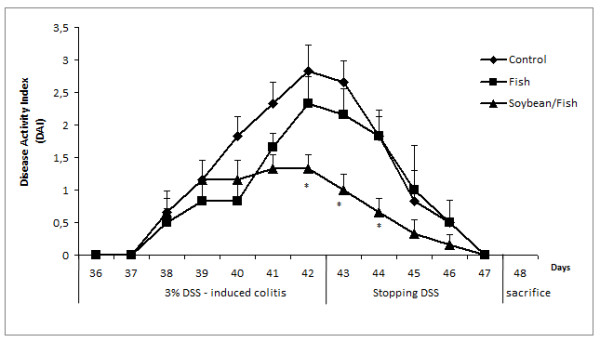
**Time course of changes in the DAI (combined scores of weight loss, stool consistency and bleeding) in rats fed control, fish or soybean/fish diet, based on Cooper et al. criteria **[16]. Values represent means ± SEM, n = 6 rats; * Different (p < 0.05) from Control group.

The induction of colitis resulted in significant changes in body weight, stool consistence, fecal blood, food intake and a worsened general status. The three groups presented a progressive DAI from the 3^rd ^day with DSS (day 38) until the 7^th ^day (day 42) (Figure 1). During colitis induction, the F group tended to be similar to the SF group in stool consistency and weight loss; however, rectal bleeding was more intense (data not shown).

**Figure 2:**
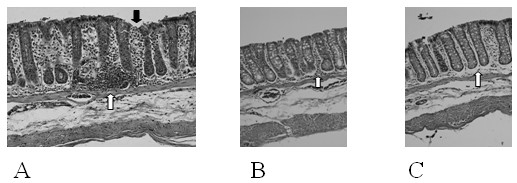
**Histology (hematoxylin-eosin, magnification, × 200) of colonic samples taken from Wistar rats receiving 3% DSS for 7 days and water for 5 days**. (A) Control group, fed control diet, showed ulceration of epithelial superficies (black arrow), intense inflammatory cellular infiltration (white arrow) and destruction of colonic architecture; (B) Fish group, fed fish diet, showed basal lamina edema and moderate cellular infiltrate (arrow); (C) Soybean/Fish group, fed soybean/fish diet showed light cellular infiltrate (arrow). The soybean/fish diet was more efficient than the fish diet in attenuating morphologic damage and preserving colonic architecture.

After stopping DSS (day 43), the clinical symptoms showed a regression, normalizing in day 47. The animals were sacrificed at day 48 (recuperation phase) without any clinical symptoms (Figure 1), although the colon damage remained as the histological examination graded by a pathologist (Figure 2).

SF group presented a significantly decreased DAI on days 41, 42 and 43 when compared with the C group. The F group had an intermediate DAI, which was between those for the C and SF groups (Figure 1).

Colon length, an inflammation indirect marker, was higher in the SF and F groups in relation to the C group (Table 4). In addition, IS revealed typical inflammatory changes in the colonic architecture (ulceration, crypt dilation, mixed cell infiltration and granulocytes) in all groups, but only the SF group presented with lower tissue damage when compared with C (Figure 2), probably associated with a decreased incidence of diarrhea, blood in feces and smaller weight loss in this group.

**Table 4:** Colon length (cm), inflammation score and corticosterone concentration (μg/100 mL) in DSS-induced colitis rats fed control, fish or soybean/fish diet.

	Colon length	Inflammation Score	Corticosterone
Control	14.26 ± 0.24	2.33 ± 0.33	14.57 ± 1.40
Fish	16.85 ± 0.30*	1.83 ± 0.30	14.63 ± 1.11
Soybean/Fish	17.21 ± 0.28*	1.16 ± 0.16*	16.17 ± 0.89

Values represent means ± SEM, n = 6; * Different (p < 0.05) from Control group.

Regarding plasma corticosterone levels, there were no differences among the groups.

### Cytokines, MPO activity and DNA damage

MPO activity was significantly lower in the SF group than in the C group, suggesting a reduced neutrophil infiltration in colon tissues, and again, the F group had an intermediate result (Table 5). Interestingly, no difference was observed in the IL-4 and INF-γ cytokine tissue concentrations. However, in relation to IL-10, an important cytokine in maintaining gastrointestinal mucosal homeostasis, increased values were found in the F and SF groups when compared with the C group (Table 5).

**Table 5:** IL-4, IL-10 and INF-y concentrations (ρg/mL), MPO activity (U/mg) in the colon and DNA damage levels (tail moment/100 cells isolated from colon) of DSS-induced colitis rats fed Control, Fish or Soybean/Fish diet.

	IL-4	IL-10	INF-y	MPO	DNA damage
Control	209.36 ± 47.30	310.01 ± 33.36	206.08 ± 37.27	0.84 ± 0.23	4.66 ± 0.16
Fish	255.66 ± 37.25	539.79 ± 57.14*	234.81 ± 21.04	0.60 ± 0.09	3.46 ± 0.11*
Soybean/Fish	272.64 ± 44.22	653.50 ± 61.69*	285.15 ± 43.35	0.21 ± 0.06*	2.94 ± 0.19*

Values represent means ± SEM, n = 6 rats; * Different (p < 0.05) from Control group.

**Figure 3:**
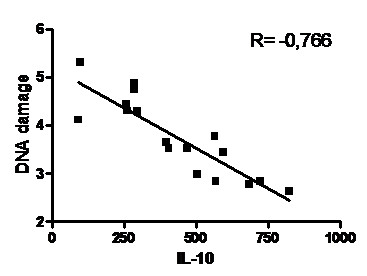
**Correlation between DNA damage levels (tail moment/100 cells isolated from colon) and tissue IL-10 concentration (ρg/mL) in all DSS-induced colitis rats**.

The detected DNA damage levels in colon were significantly lower in the SF and F groups (Table 5), suggesting an inverse association between decreased DNA damage and increased IL-10 levels. In fact, a linear correlation test between these variables was performed and a strong association between an increase in IL-10 and decrease in DNA damage (r = 0.77) was found (Figure 3).

## Discussion

The results showed that the colon inflammation induced by DSS was significantly less severe in group SF, showing that the mixture of fish oil and soybean oil balanced the w6:w3 PUFA ratio (2:1) and improved colonic inflammation. The diet enriched with fish oil (F group) presented intermediate effects, probably because of the imbalance in the w-6:w-3 PUFA ratio (1:6). Several studies demonstrated the importance of modulating the w-6:w-3 ratio to obtain beneficial effects rather than simply reducing w-6 PUFA levels [21]. The imbalance in the w-6:w-3 ratio, as is observed in western diets (10:1), may be related to an increased production of proinflammatory cytokines and eicosanoids in autoimmune diseases and IBDs [22]. Fish oil contains large amounts of SAFA, which has been associated with chronic diseases [23,24]. Several studies indicate that the optimal w-6:w-3 ratio may vary according to the disease; however, the ratio between 5-2:1 has been associated with decreased inflammation in patients with IBD, rheumatoid arthritis and other inflammatory diseases and with reduced rectal cell proliferation in patients with colorectal cancer [25].

It has been suggested that patients with IBD show changes in the metabolism of long chain polyunsaturated fatty acids (LCPUFAS). These fatty acids are well known to be parts of the cell membrane and precursors of important eicosanoids, which participate in inflammatory response. Alterations in the availibility of certain LCPUFAS can exert influence on the inflammatory and anti-inflammatory eicosanoids production [12] and may be relevant in maintaining the chronic inflammatory activity in the colon [26].

Fish oil supplementation may be able to down-regulate the expressions of some genes, which have been involved in UC [12,27].

In this paper, we evaluated animals in the recuperation phase, 5 days after stopping DSS exposure. We decided to evaluate this period for considering diet as a complementary therapy; in a severe inflammation model, the benefit effects would be more difficult to observe. The protocol was started using DSS 5% in drinking water for 7 days, and later, it was decreased to 2% for 10 days, when the animals were sacrificed [21]. However, the mortality in this experimental model was 80%, and so we decided to decrease the DSS to 3% for 7 days and water for 5 days. This high mortality could be associated with different molecular weight of DSS and/or the animals' gender. The mol wt of 36,000 - 50,000 for DSS and Wistar female rats were not used in this paper.

As expected, all animals presented rectal bleeding characterizing the colitis symptoms. It was observed that this bleeding was intensified in the F group, being associated with the higher elevated DAI found. The high w-3 intake in the F group (shown in Table 2), as compared to C and FS group, could be associated with a decreased production of thromboxanes A_2_, a potent platelet aggregator, as it has been demonstrated with fish oil rich-diets [23,25,28]. The DAI evaluation showed lower values in the SF group suggesting that the balance of fish and soybean oils exerts protective effects in decreasing disease activity and protecting against weight loss. These are important factors considering that deficit in the nutritional status occurs in patients with IBD during the disease activity and that dietary fatty acids interventions might be beneficial and improve clinical and nutritional status [29].

In this paper, increased IL-10 levels in the SF and F groups could be associated with a protective effect of diets on weight loss, colon length and DNA damage, important inflammatory factors in IBD. Cytokines play a key role in the development, recurrence and exacerbation of the inflammatory process in IBD. IL-10 is an immunoregulatory cytokine that influences the immunological system, both on the innate and cell-mediated response. It affects the gastrointestinal mucosal homeostasis through the down-regulation of colon inflammation and the inhibition of both antigen presentation and release of pro-inflammatory cytokines, and it is related to the activity of regulatory cells [30,31].

No difference was found in INF-y and IL-4 cytokines. Evaluating cytokine release in experimental colitis, Dieleman *et al*. [32] also found no increase in IL-4 and INF-γ in the acute phase of UC. These authors observed these elevated cytokines only in later phases (14 days after DSS stopping).

An increase in the colonic MPO activity, a specific marker of polymorphonuclear neutrophils activity, was used as a measure of the inflammatory status [33]. The present study showed that the MPO activity decreased only in the SF group in relation to the C group, which matched the lower inflammation based on the inflammatory score and decreased DNA damage. Once more, the balanced w6:w3 ratio used in the SF group shows a beneficial effect on UC. In fact, neutrophils may mediate a mucosal injury by the synthesis and release of ROS [6], and it has been known that oxidative stress is a pathogenic factor correlated with DNA damage [9].

There was a clear correlation between increased IL-10 levels and decreased DNA damage, and to the best of our knowledge, this is the first demonstration of a causal association between these variables associated with IBD. It is likely that the anti-inflammatory effects from IL-10 attenuating mucosal inflammation are associated with putative protection in DNA.

Considering that inflammation can accelerate tumorigenesis in the colon, and anti-inflammatory drugs have been used to prevent this event, a dietary intervention with a mixture of fish and soybean oil may be an effective complementary therapy to prevent cancer. Also, it could be an alternative to the use of anti-inflammatory drugs and their associated side effects.

Some studies have concluded that there are some benefits of fish oil in IBD [34-36] However, a recent study reported no effect of w-3 PUFA on disease activity in humans IBD [37]. These controversial results have been attributed to the different w-3 PUFA doses used [28]. Here, the mixture of fish and soybean oil in the diet was demonstrated to be better than the use of fish oil as an exclusive source of fat.

Our laboratory has demonstrated that a diet rich in fish or soybean oil could exert beneficial effects on the acute inflammation model. This effect was partially attributed to the elevated basal corticosterone levels induced by these diets [38,39]. Here, we show that plasma corticosterone levels did not differ in the three experimental groups. This strongly suggests that the anti-inflammatory effects obtained by dietary treatment using fish or soybean/fish diet could not be attributed to the action of this hormone.

In conclusion, the main beneficial effects exerted by the balance between fish and soybean oil were in reduced disease activity, improved histological score, increased IL-10 cytokine, decreased MPO and protection against DNA damage. The inverse correlation between IL-10 levels and DNA damage also demonstrated that the w-6:w-3 ratio (2:1) was important in reducing disease activity and colon cancer prevention associated with colitis.

## Competing interests

The authors declare that they have no competing interests.

## Authors' contributions

RANX and GGA were recipient of doctoral fellowship from Coordenação de Aperfeiçoamento de Pessoal de Nível Superior (CAPES). KVB, RANX and GGA performed the animal experiment, CARM performed the histological analysis, MLR performed the Comet assay of the colon samples, AG performed the MPO analysis. POC performed the analysis of fatty acids composition of diets. CMON contributed to the analysis and discussion of data, VLFS supervised the experiment, obtained funding and provided administrative, technical, and material support. KVB and VLFS analyzed, interpreted the data and wrote the draft of the manuscript. All authors critically reviewed the manuscript.
